# Automatic Target Recognition Based on Cross-Plot

**DOI:** 10.1371/journal.pone.0025621

**Published:** 2011-09-29

**Authors:** Kelvin Kian Loong Wong, Derek Abbott

**Affiliations:** 1 School of Aerospace, Mechanical and Manufacturing Engineering, RMIT University, Bundoora, Victoria, Australia; 2 School of Electrical and Electronics Engineering, University of Adelaide, Adelaide, South Australia, Australia; National Microelectronics Center, Spain

## Abstract

Automatic target recognition that relies on rapid feature extraction of real-time target from photo-realistic imaging will enable efficient identification of target patterns. To achieve this objective, Cross-plots of binary patterns are explored as potential signatures for the observed target by high-speed capture of the crucial spatial features using minimal computational resources. Target recognition was implemented based on the proposed pattern recognition concept and tested rigorously for its precision and recall performance. We conclude that Cross-plotting is able to produce a digital fingerprint of a target that correlates efficiently and effectively to signatures of patterns having its identity in a target repository.

## Introduction

### 1.1 Automatic Target Recognition

Automatic target recognition (ATR) is a technology that can isolate a target from a noisy background and perform classification of the object [1]. A reliable computerized pattern recognition system is crucial for image analysis especially when images are registered at rates higher than human-assisted visual review processes. For such applications, the time-critical identification of targets that are acquired by an imaging modality is vital.

The interest and research in ATR technology has resulted in the development of various systems for identification of targets over the past few decades. Utilization of the sophisticated synthetic aperture radar (SAR) to collect information on target vehicles may be deployed [2]. An airborne SAR takes radar soundings from the ground along a precisely measured flight path and then separates the various target locations by sophisticated signal processing. This allows the formation of extremely detailed fine-resolution maps of radar-reflectivity of a target scene. The SAR image data is analyzed for feature modeling. Each target image has its own unique signature for use in ATR algorithms.

Concepts in designing and implementing ATR algorithms include signal and image processing, target detection, isolation and segmentation, motion analysis and tracking, statistical or model-based recognition, and signature modeling. An ideal shape pattern recognition system has an inbuilt identification algorithm that can fully recognize targets without human intervention and with low false alarm rates [1]. This performance is required to be relatively robust to sensor noise and target orientation. The problems in existing methods are the heavy computational overhead in identifying targets rapidly, while taking into account all the filtering and pattern normalization procedures.

In ATR systems, the key operational procedures are target detection, discrimination and recognition, and performance assessment. In this paper, the emphasis is on the second and third procedures. The aim is to reduce computational expense for this purpose, and the objective is to develop an operational system that addresses this issue when predicting target identity. Here, the Cross-plot [3], [4], [5] is proposed to address the aforementioned problems in pattern recognition and to identify the target of interest. This study highlights the conceptual development of the technique and performs proof-of-concept experiments.

### 1.2 Feature Extraction and Classification

We can identify two binary objects according to the difference between their features. In pattern recognition, features are extracted from a target image containing distinctive information.

**Figure 1:**
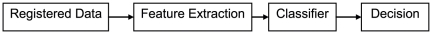
Operational stages of a pattern recognition system. The flow chart of recognition processes can be illustrated using four simple stages: registration of data, extraction of the target's features, classification, and decision making. The feature extracted from the registered data is fed into a classifier, which can be based on Cross-plot, artificial neural network or Bayesian network, for target object classification. The accuracy of identification is dependent on the quality of features being extracted. Finally a decision is made based on the classified results that will lead to the identification of the interested target.

Choosing discriminating and independent features is the key to any successful pattern recognition system. It is usually difficult to judge a registered object of interest by acquiring raw data related to it as the recorded information is usually noisy. Therefore, the raw data must be transformed into a reduced set of features to be used by the classifier. A process of mapping the original measurements into more effective features is generally known as feature selection or extraction (Figure 1). The low dimensional features contain sufficient relevant information to avoid the problem of classifier over fitting.

For a given set of signal features that have been extracted, an appropriate classifier needs to be trained by utilizing part of the data and the known corresponding labels for fitting data into feature space. Specifically, the training data is used to populate the hypothesized clusters of the training data, and the built clusters can represent the classes in the feature space. Typically, the data is divided into two parts: one for training and the other for testing in order to verify the classification capability. Generally, it is assumed that the training and testing data have similar properties and distribution, which is a pre-condition of feasibility in classification.

### 1.3 Shape-Based Image Retrieval

Image-based target recognition has been developing rapidly in past decades. Shape-based image retrieval [6], [7], [8] is one of the most popular methods and inspires the proposed Cross-plot technique. The shape-based image technique lies within the shape analysis and feature extraction paradigm. In shape analysis, complex spatial features of binary images are represented using their linear approximations, and easing the computational burden for carrying out matches between spatial objects. Shape pattern representation techniques can be divided into two categories, namely, the boundary-based and region-based methods [9]. Both methodologies can be further sub-categorized as the transform and spatial (geometric) domains, depending on whether direct measurements of the shape are used or a transformation is applied [10]. Feature extraction is the process of gaining geometrical information from a shape that has the location, scale and rotational effects filtered out from it. The resulting feature vector will be a pattern representation of an exact geometry of the spatial object. Shape-based retrieval takes into consideration issues such as robustness and stability of the various shape representation techniques. Successful retrieval systems have good matching abilities for shape objects that are subjected to distortion, scaling, translation, noise, and region loss when using the same feature set. Therefore, the selection of a feature extraction technique is critical for achieving high recognition performance [11].

#### 1.3.1 Region-based Methods

Region-based techniques extract information regarding the internals of the shape besides the boundary details. Some of the more established methods utilizes the Zernike [12], pseudo-Zernike moments and wavelet moment invariants [13]. Transform-based methods encompass the Hough transform and spatial-based methods take into account geometrical measurements of the shape's fundamental characteristics. Shape-based image retrieval is performed using a feature vector comprising of the solidity (S), eccentricity (C), and extent (X) of shapes, which form the SCX feature set [14]. The choice of these shape measures is not a conditional necessity. There are many other features such as shape compactness, aspect ratio, holes, rectangularity, max-min radii, elongation, symmetry, circularity, and Euler number that can be used [10]. The Query By Image Content (QBIC) system by International Business Machine (IBM) uses statistical features to represent the object shape [15]. Its feature set includes area, circularity, eccentricity, major axis orientation, and algebraic moment invariants. The Hough transform [16] has been extensively used for shape detection and recognition. Specific requirements of geometric invariance, storage and computational complexity, as well as support of appropriate similarity measures are considered.

#### 1.3.2 Boundary-based Methods

In boundary-based image retrieval, methods using chain codes [17], [18], [19], polygonal approximations [20], Delaunay triangulation [21], Fourier descriptors [9], [22], [23], [24], boundary moment invariants [15], [21], [25], and two-dimensional (2D) strings [26] can be deployed. Boundary-based image retrieval uses only the contour of the object shape and ignores the region in the interior. The System for Trademark Archival and Retrieval (STAR) [27], [28] uses features based on invariant moments and Fourier descriptors extracted from manually isolated objects. A spatial-based object retrieval technique that is a sub-category of the boundary-based methods such as the Touch-Point-Vertex-Angle-Sequence (TPVAS), Bounding Circles (MBC) and Angle-Sequences (AS). A retrieval architecture based on MBC utilizes three different structures on features that are extracted from the objects' MBC. The Hausdorff distance between planar sets of points is known to be an effective measure for determining the degree of resemblance between binary shape patterns [29], [30]. For chain codes, the boundary of a binary image is traversed and a string representing the curvature is constructed. A shape can be converted into chain information representing the boundary. Transformation-based approaches can be further broken down into two sub-categories: functional and structural [31]. Functional transformations such as Fourier descriptors to structural transformations, such as chain codes and curvature scale space feature vectors, comprise some of the transform-based methods. A comparison of the performance for Fourier descriptors, chain codes, Delaunay triangulation, and TPVAS that are used in shape representation and retrieval of scaled, rotated and translated shape pattern variants is studied [10]. Pattern recognition based on shape context relies on the normalized spatial distribution of landmark coordinates from shape contours [11], [30], [32], [33]. For this method, the shape of an object is essentially captured by a finite subset of its points. Distribution of shape part is based on a reference point relative to it, thus offering a global discriminative characterization [34]. However, some issues exist such that the reference points taken on a discontinuous contour of an incomplete shape with significant loss of regions may result in poor same characterization. Other techniques such as the inner-distance, which is defined as length of the shortest path between landmark points within the shape silhouette, can be incorporated into shape context and is specialized to identify dissimilarity in patterns that may be similar in spatial distribution but dissimilar in part structures [35], [36]. Moreover, shapes parts may be disconnected and renders the inner-distance method unreliable. As a result, identification of region-loss shapes will not be optimal as their correlation indices will be highly dissimilar.

### 1.4 Cross-plot Based Target Recognition

One of the important breakthroughs in the early work of computer vision research is the recognition of a two-dimensional pattern as a perspective view of the three-dimensional scene of objects [32]. From the technical viewpoint, capturing the silhouette of the object as a shape pattern and generating its Cross-plots unique to that pattern can be achieved. At the present stage, the notable concept is the introduction of strategically positioned reference nodes, thereby generating Cross-plots of each of the individual node and the feature points representing the target pattern. This modification contributes significantly to the success of the technique in recognizing binary silhouettes of visually captured air targets. Cross-plot signature generation satisfies the characteristics of automatic target recognition. Performance verification was carried out by assessing the robustness of target recognition to defective imaging based on a catalogue of patterns with incremental degrees of deviations (Appendix S1). Performance calibration is based on a repository of target images (Appendix S2) and system validation is achieved by real-time air target recognition from a documented video database.

It is impractical to extract every single feature of a target pattern with high precision while ignoring the noise in the background. As such, only the vital features of a pattern should be selected for the signature design in order to achieve an effective pattern differentiation. The proposed technique is able to extract the significant shape content of the pattern for signature formation with minimum pre-processing of the image and no prior normalization of the target size. Details of the proposed technique based on this Cross-plot concept are shown in the following sections.

## Methods

### 2.1 Definition of Cross-plot

We briefly introduce the core mathematics related to the proposed Cross-plot signature generation technique. The Hausdorff fractal dimension [37] is the fundamental Cross-plotting. Denote *D* as the fractal in a state space for a given pattern of points. The space is divided into grid cells of dimension *r*. Here, *N*(*r*) denotes the number of cells that are penetrated by a specific set of points. The box-counting fractal dimension *D* of a fractal, by counting the number of cells that contain one or more of its points, is given by
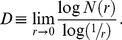
(1)


The Cross-plot [4] is a graphical representation of the Hausdorff fractal dimension, and is defined as a plot of the logarithm of the number of pixel object pair counts that do not exceed a specified proximity distance versus the logarithm of this variable proximity distance. More formally, the Cross-plot between two binary pattern data sets *A* (with *N_A_* pixel points) and *B* (with *N_B_* pixel points) is
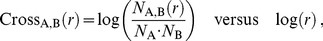
(2)where *N*_A,B_(*r*) is the count of pixel object pairs within a distance *r*. The specified proximity distance can be normalized by dividing the distance *r* with the maximum object pair distance.

### 2.2 Features by Cross-plotting

Cross-plots are generally used to identify interesting patterns in complex data sets. The notion of the Cross-plot is defined in terms of relative distance between two types of point sets. The objective is to extract the location information about the pattern by judiciously placing reference nodes around the data set and computing the Cross-plots with respect to these node points one at a time. The Cross-plot curves can be presented in the same signature graph. This section demonstrates that Cross-plot of different binary target feature sets results in distinct signature graphs and has the potential to be used for pattern recognition.

To simplify the analysis, an arbitrary number of eight reference node points are positioned around the binary pattern image in a circular manner. The radius of this circular arrangement is taken as the distance from the center of the boundary, encapsulating the binary target to any one corner of it. Cross-plots can be created with an arbitrary number of coordinates depending on the resolution. In all the examples, an arbitrary resolution of thirty-one coordinates is fixed for each curve in the family of Cross-plots. Note that *dist* denotes the distance between two data points; and *count-of-pairs* is the number of point-to-point pairs that are equal or smaller than *dist*. A description of Cross-plot generation can be found from steps 1 to 3 in Section 3.1 and the detailed pseudo-code can be referenced from Appendix S3.

Each curve of the set of Cross-plots demonstrates registration of feature point counts through the circular pattern in the region just before the plateau of a Cross-plot curve. The plateau region is the location where no new data points are accumulated after the expanding radius reaches the end of the data pattern. The growth in the accumulation of points decelerates sometime after the middle of the pattern – the count rate of feature points representing the pattern encapsulated by the boundary extended from a node of interest decreases.

**Figure 2:**
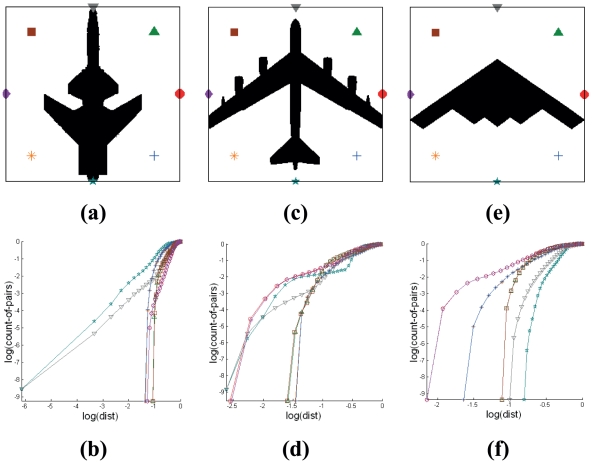
Effect of pattern target variants on Cross-plots based signatures. Images (a, c, e) are three different binary patterns with eight reference nodes positioned around their feature point data sets; and (b, d, f) are their corresponding Cross-plot signatures respectively. Binary images of a silhouette target that are generated from acquired images with variations in target roll, pitch and yaw (from a two-dimensional visual perspective) are presented in (g, i, k). The images (h, j, l) show the Cross-plots of binary patterns.

Every unique pattern will yield a unique set of curves for Cross-plots of the pattern with nodes positioned around it. Therefore, a set of curves for a particular binary image representing a target can be used as the signature or thumbprint of that object. Figure 2 gives some examples of the Cross-plots corresponding to three arbitrary binary targets.

For a corresponding Cross-plot signature, there are an arbitrary number of eight graphs based on number of reference nodes (*N* = 8) that are all superimposed onto a single set of axes. Each node point is able to capture the details of the pattern from a different spatial perspective. The placements of nodes around the pattern result in the curves represented by a family of plots. Note that the chain of reference nodes is superimposed onto a bounding square box, which may not indicate the minimum encapsulation of the target silhouette.

The technique of characterizing patterns in a data set by using Cross-plots has been previously studied [5]. In an attempt to detect cluster locations for a given geometrical pattern through Cross-plot analysis, certain interesting observations have been made. Cross-plots of different patterns (with every reference node point) will yield curves with varying curvature depending on the distribution of the data set under specific configurations. For instance, the number of plateaus is related to the number of clusters in the data set. Based on such observation, the uniqueness of the Cross-plot that is obtained for every specific pattern and node is dependent on the characteristics of the data set.

The unique features of a particular shape can be extracted by computing a set of Cross-plots based on a set of reference nodes with the shape pattern and representing them simultaneously using the same graph. Every unique pattern will result in a set of Cross-plots that describe the pattern characteristics. Despite that, it may be possible for corresponding sets of Cross-plots to be identical even based on different shapes, and resulting in a wrong identification. This problem can be solved by designing a new arrangement of node points spatially.

### 2.3 Identification Metric

Detection and identification of a target is based on the outcome of comparison or similarity between signatures. The signatures of some images of the observed target and the reference target have a strong correlation due to the high similarity in their family of Cross-plots. Application of the algorithm will output the shape contrast index. A high correlation means a successful match and the identity of the reference target is reported.

The similarity metric is usually defined via a distance measure that will be used for a nearest neighbor match in the feature space. Various dissimilarity measures such as the Minkowski or *L_λ_* metric [38], the Cosine metric [39], and the Hellinger metric [40] can be used in matching two sets of data points. The Minkowski *L_λ_* metric for the case of *λ* = 1 has been chosen for comparison of signatures as it is able to simply differentiate the dissimilarity of two signatures effectively. The Shape Contrast Index (SCI) is simply the sum of all the *L_λ = 1_* distances. It is inversely proportional to the similarity of the two patterns for shape comparison. With this similarity quantification, the proposed feature extraction technique is able to convert two-dimensional features into one-dimensional scalars for comparison.

### 2.4 Invariant Properties of Cross-plot

The Cross-plots are generated from the binary pattern image. Therefore, the various distortions on the binary pattern image will affect the generated corresponding Cross-plots. Ideally, the binary pattern image is pre-processed from the original two-dimensional image to reduce various distortions. However, this is not always practical. This section discusses the effects of pattern deviations, which are sampled from the catalogue of images in Database B (Appendix S2), on the corresponding Cross-plots of target patterns.

#### 2.4.1 Distortion

**Figure 3:**
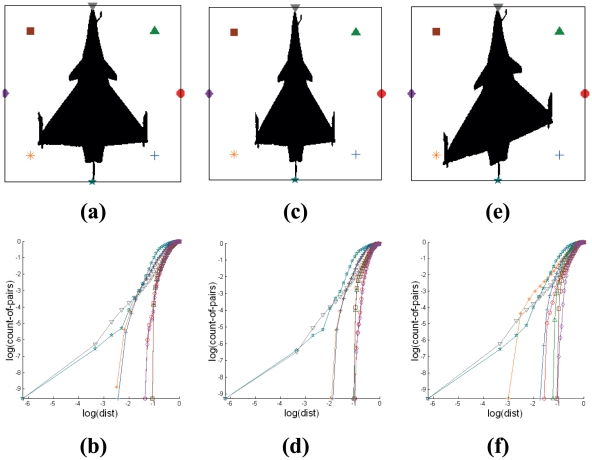
Effect of binary pattern with shape distortion and Cross-plots based signatures. The graphs demonstrate that the variation of Cross-plots can be attributed to the slight distortion in target patterns. The differences presented by the shape contrast indices of these variants are relatively insignificant.

This section describes how the shape distortion affects the Cross-plots. Figure 3 shows three targets with the same structure but mapped onto different projections and their corresponding Cross-plots. Assume the shape in Figure 3 (a) is the reference shape, target in 3 (c) is spatially compressed to 75% of its original size in the horizontal orientation and target in 3 (e) is sheared vertically at an angle of 20°. As can be seen, the difference between the binary patterns Figure 3 (a), (c) and (e) are detectable, however, the change in their Cross-plots is small. The SCI of Cross-plots for Figure 3 (b) with (d) and for Figure 3 (b) with (f) are 10.3 and 15.6 respectively (Refer to Section 2.3 for the definition of identification metric used in here). This shows the relatively low sensitivity of Cross-plots to a certain level of shape distortion.

#### 2.4.2 Target Mirroring

**Figure 4:**
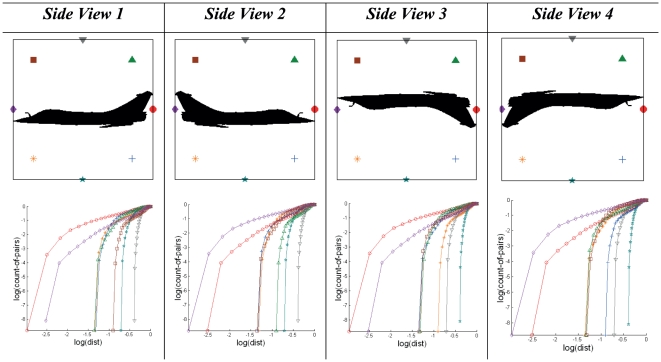
Effect of target orientation on Cross-plot based signatures. Pattern images representing aircraft target silhouettes based on four different views and their respective Cross-plots. The orientation of the target generates Cross-plots with similar curvatures but having different positions exist in the same signature set.

Generating Cross-plots using an encapsulation of nodes that are arranged uniformly around the pattern boundary results in graphical representation that are identical irrespective of the same target pattern taken from different isometric views of the same visual plane. The sequence of nodes taken to generate Cross-plot curves is different for different views. Figure 4 demonstrates four symmetrical views (vertical and horizontal) of the same target and their corresponding Cross-plots.

Each reference node point that is Cross-plotted with the pattern produces a curve (each being represented by using a different symbol). Difference in arrangement of the pattern with respect to a set of node points will result in the same corresponding curve to be represented by different symbols. If the node at the nose of the aircraft is taken as the start node point for Cross-plotting with the entire target pattern, the signatures produced from each of these node points will be similar for all four target orientations. The SCI of Cross-plots for side views 1 to 4 is derived to be 3.00. As the number of node points in the circular encapsulation increases, the variation of angle of orientation of the pattern in affecting the signature decreases.

#### 2.4.3 Target Pitching

**Figure 5:**
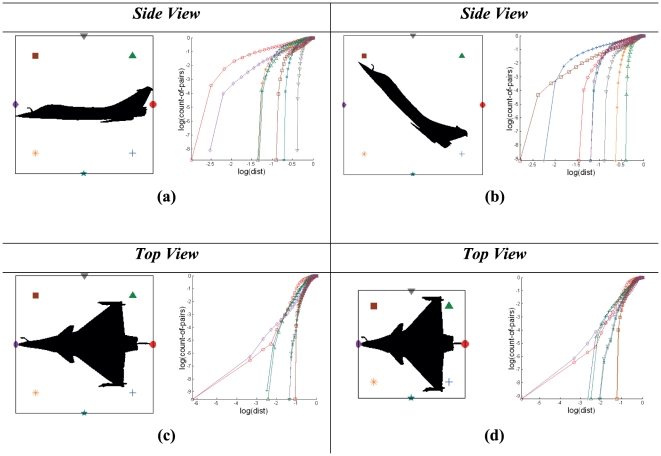
Effect of silhouette targets pitched at 0° and 45° on Cross-plots based signatures. The orientation of the target generates a different pattern based on its silhouette onto a two-dimensional plane. The signatures based on the Cross-plots that pertain to every target are shown to have small variations.

Different orientations of the three-dimensional object produce different orthographic views when they are superimposed onto a two-dimensional visual plane during monitoring (Refer to Figure 5). Figure 5 (a, c) shows the corresponding signatures for images of the fighter jet pitched at 0° and Figure 5 (b, d) illustrates that for target silhouette pitched at 45°. For the case of side view of the target in Figure 5 (a, b), we determine the boundary of nodes encapsulating the target pattern using the Minimum Boundary Circle (MBC) method [14]. The displacement in angular orientation of the object with respect to a specific axis results in the same graph that is represented by different ordering of symbol representing the nodes. Rotating the arrangement of the nodes using the same angle as the pitch of the aircraft reduces deviation in its signature. The SCI of Cross-plots for Figure 5 (a) with (b) and for Figure 5 (c) with (d) are 7.1 and 6.9 respectively (Refer to Section 2.3 for the definition of identification metric used in here).

#### 2.4.4 Scaling

**Figure 6:**
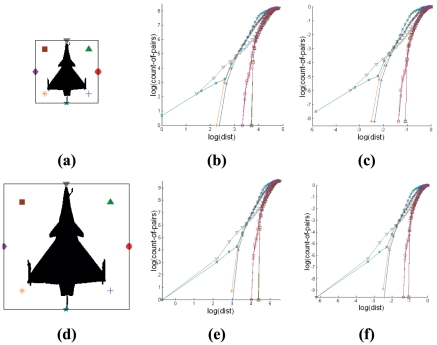
Effect of scaling of shape pattern on Cross-plots based signatures. The subfigures are as follows: (a) Binary image at resolution of 128 by 128; (b) Cross-plots of binary image at resolution of 128 by 128; (c) Cross-plots of binary image at resolution of 128 by 128 with encapsulations spanned by distances that are normalized; (d) Binary image at resolution of 256 by 256 without normalized spanning distance; (e) Cross-plots of binary image at resolution of 256 by 256 without normalized spanning distance; (f) Cross-plots of binary image at resolution of 256 by 256 with encapsulations spanned by distances that are normalized.

The Cross-plots retain their curvature integrity despite scaling of the resolution of the target pattern (Refer to Figure 6). The scaling for a typical image in multiples of two is performed once (Figure 6 (a, c)) and the corresponding Cross-plots are computed in Figure 6 (b, d). The scaling of pattern resolution will cause a very slight shift in the logarithmic distance axis of the Cross-plots, without any change in their curvatures.

The Cross-plot extracts information from the pattern such as the number of feature points in the data set encapsulated by a radial boundary, from a stationary reference node point, in the two-dimensional Euclidean space. The number of points relative to the image size is similar for both resolutions, if twice the radial extension from the node is taken for the 2 times resolution image. Based on this observation, obtaining the signature of a pattern image at *n* times resolution can be achieved by a shift of magnitude of log(*n*) for the logarithmic axes of the Cross-plots for the given 1 time resolution binary pattern, instead of applying computationally expensive spatial quantization to the image before signature generation as shown in Figure 6 (c) and 6 (d). Normalization of the number of counts of the pixels and the radial scanning distances for any specified image resolution, as demonstrated in the Cross-plot formula, is equivalent to a shift of the axes by log(*n*). Figures 6 (e) and 6 (f) illustrate that Cross-plots for the same pattern of different dimensions will be similar after normalization. This is in contrast to the Figures 6 (b) and (e), which represent non-normalized count-of-pairs and radius spanned for the pixel counts.

#### 2.4.5 Noise

The main sources of degradation are sensor noise, and the scattering and attenuation of electromagnetic radiation by atmospheric particles in an intervening propagation medium, resulting in fuzzy target boundaries [41], [42]. This type of noise can affect the binary image pattern greatly. However, the noise on the binary image pattern can be mitigated using the Cross-plot technique because the fine details can be smoothed and then reduced in resolution.

**Figure 7:**
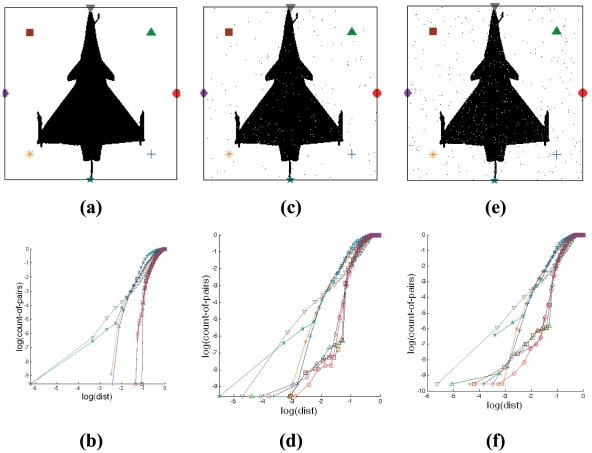
Effect of noise on Cross-plot based signatures. (a) Binary pattern of target without noise; (c) Binary pattern of target with SNR of 23dB; (e) Binary pattern of target with SNR at 20 dB; (b, d, f) are the Cross-plots of patterns from (a, c, e) respectively.

For simplicity in our experiments, we generalize noise using a randomly generated normal distribution. The level of noise is denoted by the signal-to-noise ratio (SNR), where the SNR is defined as the contrast of object divided by the standard deviation of normally distributed random noise. The unit of this measurement is the decibel (dB). Figure 7 shows the Cross-plots of images with three different signal-to-noise ratio (SNR) levels. Figure 7 (a) is a binary image pattern without any noise; Figure 7 (c) is a binary image pattern with SNR of 23 dB and the SNR in Figure 7 (e) is 20 dB; Figure 7 (b),(d),(f) are the corresponding Cross-plots of Figure 7 (a),(c),(e) respectively. As the SNR decreases, the possibility of correct target recognition decreases accordingly. Therefore, the level or threshold of SNR to which this technique is robust relates the accuracy of correct target recognition to be achieved.

#### 2.4.6 Part Discontinuities

**Figure 8:**
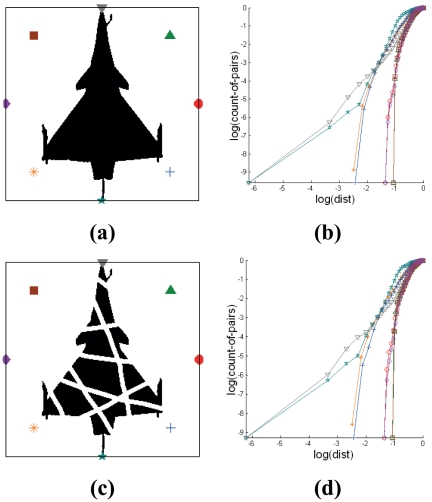
Effect of pattern defects on Cross-plot based signatures. Comparison of signatures (b,d) generated from clear (a) and noisy (c) images. The subfigures are as follows: (a) Binary pattern of target without region loss; (b) Cross-plots generated from pattern without region loss; (c) Segregated binary pattern of target due to region loss; (d) Cross-plots generated from segregated pattern due to region loss.

The existence of severe noise in an image may result in the disconnection of the pattern into segments but with the overall shape being retained. The effect of pattern truncation and segregation is investigated based on such a defect (Refer to Figure 8). Due to the overall statistical contribution of pixel counts from the complete regions of the pattern, the destruction of counts from the missing regions will not modify the general characteristic of the Cross-plots. The aggregation of pixel counts for each scanning distance is affected slightly and retains much of the inherent pattern information. This can be seen from the SCI of Cross-plots for 8 (b) with (d) that has a value of 3.20 (Refer to Section 2.3 for the definition of identification metric used in here).

### 2.5 Standardized Alignment of Targets

**Figure 9:**
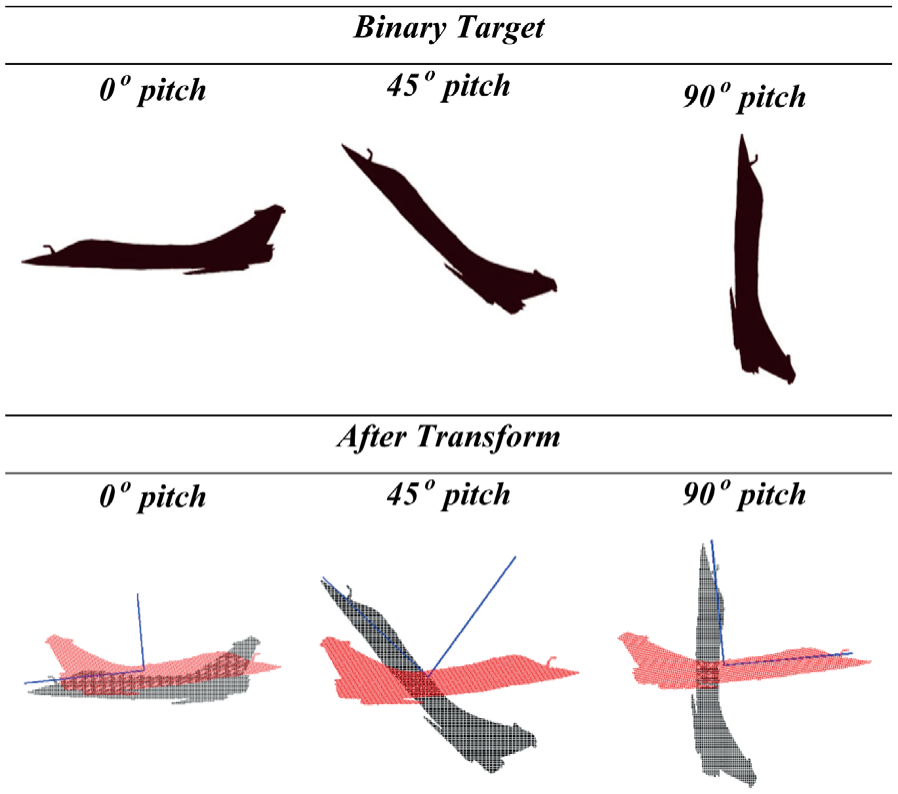
Alignment of patterns along their most prominent principal components. The above observation demonstrates that all variants of targets at different orientations can be mapped onto a base pattern, which fulfills standardization of target alignment. This will enable a standard set of reference node points around the binary targets.

This section explores the concepts behind Singular Value Decomposition (SVD) [43], [44], [45] and demonstrates how SVD can be applied onto a pattern data set to enable mapping to a standard orientation. The result map can serve as a base pattern for all target variants of different orientation. The previous observations show that the Cross-plot signatures may have similar curvatures that are generated by nodes at dissimilar positions. To solve this problem, the concept of principal component analysis is applied. Figure 9 illustrates that the most prominent principal component can serve as the directional alignment of the target and presents the reconstructed pattern along this component. The reconstructed targets for all the variants are identical and serve as the standard base pattern for which their signatures will be generated for identification or database storage in the retrieval system.

### 2.6 Verification based on Pattern Variants

**Figure 10:**
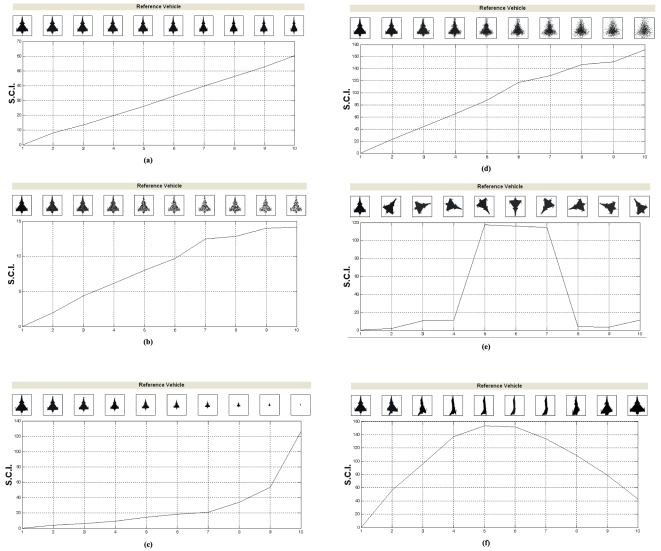
Measurement of shape contrast based on its pattern variations. The sub-figures that represent Shape Contrast Index (SCI) variations of pattern variants with respect to a reference pattern are as follows: (a) Correlation of distorted variants; (b) Correlation of pixel-lost variants; (c) Correlation of scaled variants; (d) Correlation of blurred variants; (e) Correlation of rotated variants (rotation in a two-dimensional plane about an axis); (f) Correlation of disoriented variants (target displacement about roll axis in three-dimensions and captured as a binary image from a two-dimensional perspective).

To quantify the degree of variation in shape and size, images of binary patterns in Appendix S1 illustrates various degrees of structural and dimensional distortions. Figure 10 presents some of these deviations from the original pattern and their corresponding SCIs. Under each category of pattern deviation, the SCI of corresponding modified binary image is computed and compared. The first image along horizontal axis in each subfigure is the original pattern image (or reference image). The values on the vertical axis are the SCIs of their corresponding images. The system quantifies the degree of pattern variation when correlated with a standard model, i.e. top view capture of a target that is the first pattern on the left of the images. The degree of contrast varies incrementally as the variant patterns distortions amplify in incremental stages. This allows the model to detect the degree of contrast for the input pattern variant as it varies with the level of distortion or orientation.

The effect of horizontal distortion is investigated (Figure 10 (a)). The images following reference image are horizontally compressed with a step 5%. In Figure 10 (b), the effect of pixel loss is investigated. The pixel loss is performed by scattering random white pixels on the pattern image. In this simulation, it is to add noise to the image pixel. For the successive images following the reference image, the noise is increased by 0.4 dB for each image. In Figure 10 (c), scaling is performed by decreasing the size of both vertical and spatial dimensions by an interval of 10% for each step. In Figure 10 (d), the effect of blurring is accomplished in the spatial domain by pixel averaging in a neighborhood which is known as sharpening in term of image processing [46]. The degree of sharpness changes with the radius of the neighborhood to be averaged. Images shown in Figure 10 (d) are 256 by 256 pixels. The sharpness of the successive images following the reference image is adjusted based on the change of sharpening radius with an increase by 2% of the image width. In Figure 10 (e), each image after reference image is clockwise rotated on the same plane at angular increment of 36°. In 10 (f), the target is rotated along its roll axis with an angular displacement of 20° on a single plane. Note that this set of images based on three-dimensional rotations is from Dassault Aviation [47].

The SCI indicator of the query patterns is affected by increasing scales of distortions for shape object changes based on a set of ten frames. The effectiveness of the shape-based representation model can be gauged by its recognition of variant patterns based on a different geometric feature for each target set. The whole procedure shows that under a controlled experimental setup, the increase in the SCI value corresponds to an increase in the variation of the reference target pattern. The smoothness of the correlation curve demonstrates the sensitivity of the model when responding to incremental modification for variant geometrical patterns.

## Results

### 3.1 System Methodology

A computational prototype of the automatic target recognition (ATR) methodology based on the proposed Cross-plot technique is developed. This section provides the details of the system that is implemented to match a target signature to an identical one in the database. For an appropriately tested user-defined number of node points and resolution of the Cross-plot signature, target identification based on a repository of 60 targets in real time is reliable and achievable within time in the order of milliseconds.

To generate the signature of the target, the raw image must be pre-processed to remove noise and to segment all the objects in the image for identification. The outline of the target, in terms of structural details in the image, has to be extracted accurately. The following procedures provide the guidelines for image preprocessing. For preprocessing of images of targets with distinct demonstrated features, the image is converted into a binary form through amplitude quantization. For 2D feature space, such as an image, the process of representing the amplitude of the 2D signal at a given coordinate as an integer value with *L* different gray levels is usually referred to as amplitude quantization or simply *quantization*. The effectiveness in capturing the silhouette of the aircraft for images depicting a target with an evenly shaded interior and a background that differs in contrast to a specified degree varies.

To extract the targets from low contrast images where the gradient magnitude of the target and background is in the intermediate range, utilization of edge information is indispensable [42]. Segmentation of different objects in the image can be achieved by performing image segmentation such as connected component labeling [48], [49], [50]. For less distinct images of targets whose intensity contrast with the background is low due to light reflection, segmentation techniques such as Markov random fields [51] and watershed segmentation [48] may be applicable for segregation of different objects in the image based on intensity and proximity.

Based on the understanding of the theory and observations of the Cross-plots discussed in the previous sections, the pseudo code of our method is formulated. A more detailed pseudo-code can be referenced in Appendix S3 to further breakdown the following steps.

1. Remove outliers and noise surrounding the key target. Construct a boundary encapsulating the binary target. Determine the dimensions of the circular boundary to be encapsulated around the target. The radius of the circle is taken as the distance from the center of the rectangle to any one corner of its boundary.
2. Position an arbitrary number of node points uniformly and circumferentially around the circular boundary encapsulating the binary target.
3. Obtain the set of Cross-plots for *α* = 1 to *N*, 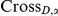 where *D* is the binary pattern points and *α* is the current node of interest from a set of *N* reference node points. The signature of the pattern is represented in a matrix that represents coordinates of Cross-plot curves that are concatenated column-wise.
4. The signatures of items in the database are compared with the target signature to give an indicator for degree of shape contrast. To compare two signatures, piecewise elemental differences in the two matrices are summed to determine a Shape Contrast Index (SCI) value.
5. The database item pertaining to the signature with the lowest SCI is returned as the identified target. Shapes with subsequent increment in SCI can be output for reference and analysis. If the SCI of all the items exceeds a user-specified threshold, reporting of an unidentified target will be returned.

### 3.2 System Prototyping

In this section, the design principles of the automatic target recognition system are described in detail and the functionalities of the system such as the actual image indexing, i.e. the computation of pattern signatures and the quantification metric for the perceptual similarity between two snap shots of the target are implemented and analyzed. These are typically off-line operations. For this system, the procedure of signature differentiation with respect to the chosen signature and metric is of lower complexity as compared to the image indexing. The overall complexity is dependent on the specificity of the signature and conservation of the spatial arrangement of the pattern. This method generates low-level signatures efficiently, capturing only the global content of the pattern irrespective of pattern distortion or target disorientation that affects it locally. The implementation and testing of the pattern indexing and recognition demonstrates the efficiency and effectiveness of our target recognition system.

#### 3.2.1 Schematic System Layout

This section briefly describes some of the working functionalities of the ATR system and how units can be integrated to form a classification system. It harnesses the Cross-plot in pattern recognition to enable an efficient and effective way of identifying airborne targets. The signature reflects the geometrical distribution of the image without executing sophisticated image processing or extracting excessive information from the shape pattern content. As a result, the computational load in generating a target's signature is low and to achieve real-time target identification is technically possible.

**Figure 11:**
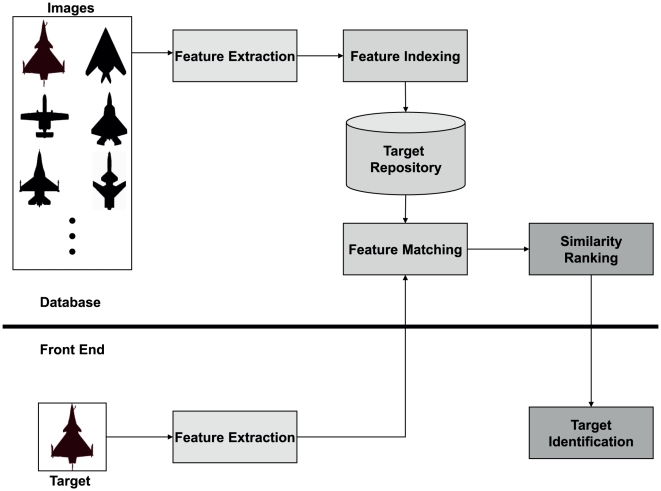
Retrieval and classification procedures for target identification. This framework lays the architecture for the development of automatic target recognition system. Key advantages of this model are that the signature generation does not require heavy computational resources, and the accuracy and speed of matching can be adjusted by the user.

The identification of a target can be achieved by the find-and-match procedure (Figure 11). The speed of matching depends on the size of the signature, and its low complexity. The implemented pattern identification and retrieval can be adjusted for speed at the expense of accuracy and vice versa. This flexibility allows for customization of the system based on the type of pattern types and size of its image library. Based on the design of the system outlined in this section, a prototype of the machine that performs target recognition is built and analyzed for its performance. Note that sampled images are from the target repository in Appendix S2.

#### 3.2.2 System Implementation

A prototype of the automatic target recognition system was built with the desired level of interactive user control and tested on a platform with different pattern registrations. The Shape Contrast Index (SCI) of the reference shape pattern with respect to the actual pattern is calculated and the patterns are well-matched based on their SCIs. The signatures of the target patterns are displayed graphically for reference. We reduced the size of the library database and use only selected input patterns of different air targets. Our main objective is to illustrate how different shape targets with high perceptual similarity may be identified. The degree of accuracy can also be displayed along with the matched target. The objective of the different experiments in the following sections and which relies on a small system database of samples is for demonstration purposes. A more reliable system can be developed by increasing the (i) database of target variants, and (ii) resolution of signatures, which can be customized to achieve the best pattern recognition efficiency.

**Figure 12:**
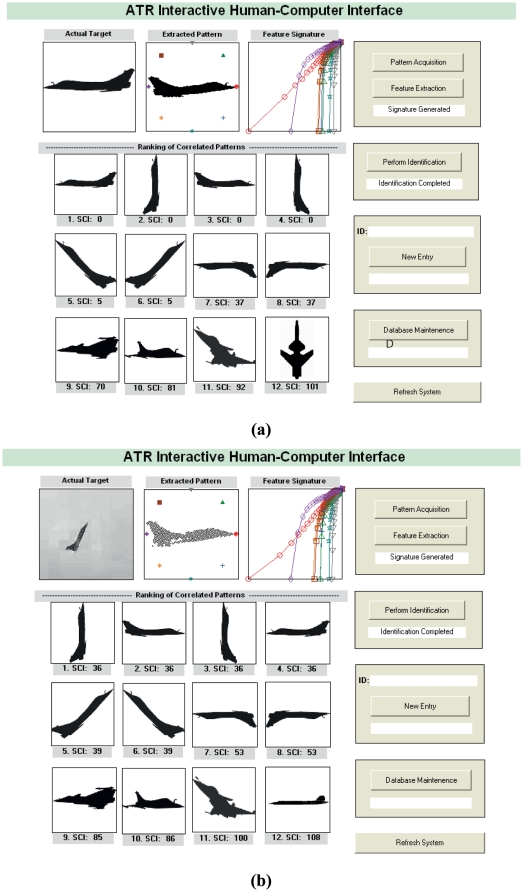
Programmable human-computer interface template for automatic target recognition system. Data for (a) Synthetic shape pattern as the identified target and (b) Photorealistic image that depicts actual target are recorded. The interface system shows that it is able to effectively identify targets with fuzzy outlines and discontinuous interiors.

Users import the digital patterns and perform feature extraction in the indexing process to generate unique signatures. There are options for entry updates into the multimedia database for matching and identification of patterns. The pattern images in the database are ranked according to their similarity with the query pattern and with their corresponding Shape Contrast Index (SCI) label. The automatic target recognition prototype is implemented and tested for reliability. In this experiment, a synthetically generated target from the side view is loaded and identified using a target library of one hundred and eighty reference patterns as shown in Figure 12 (a). In the case of a video capture of the same target, the shape object has been segmented for analysis and very similar result is obtained as observed in Figure 12 (b). No prior processing techniques to clean the image or improve the outline of the target shape are carried out.

From the ranking and correlation values, the similarity of the target and the target library data set A (in Appendix S1) can be quantified and a module has been implemented to predict the probability of its correct identification. Series of designed experiments that are based on real and synthetically generated patterns are performed with the target recognition trials, and have demonstrated robustness, accuracy, and the system suitability in the context of target recognition.

### 3.2 System Testing and Performance

In this section, the proposed technique was tested on a real data set and analyzed for effective performance and efficiency. Sampled images from a multimedia file [52] was used to create Data Set C and the thumbprints of an air target (Dassault Rafale) are provided for testing and demonstration.

**Figure 13:**
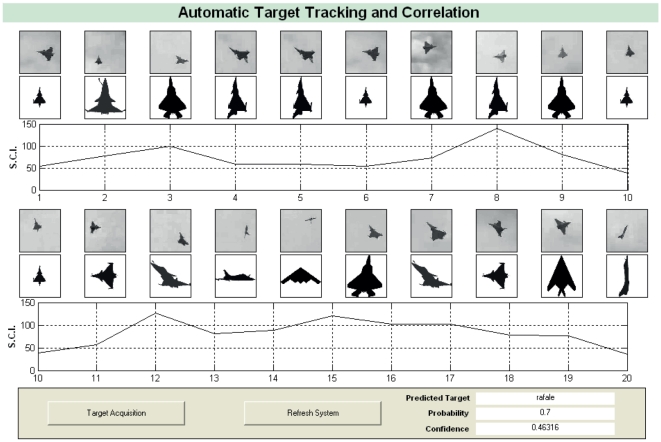
Correlation analysis of an airborne target based on acquired video clip. The data registration and feature extraction of a Dassault Rafale from a video database can be performed in real-time to consolidate sufficient content information of its shape. The time taken to verify its identity is in the order of milliseconds. The Shape Contrast Index (SCI) versus time graph demonstrates the variations in these indices as each image is captured per time frame increment. The probability and confidence of identification are output for decision makers.

The system is able to detect targets, and predict the accuracy as well as confidence of identification based on statistical analysis of SCI of every frame capture. The calculated SCI for a frame is inversely proportional to the confidence of matching. Figure 13 shows the SCI generated for every frame in a graphical format. Although our proposed technique can handle fuzzy, noisy, distorted or incomplete patterns at varying degrees, the poor image resolution of target and lack of appropriate preprocessing can affect the system accuracy to a certain degree.

A well-established pattern recognition technique by artificial neural network (ANN) was used to validate the performance of Cross-plot based ATR. The extracted features are dependent upon the structure of the segmented target and based on a set of standardized invariant moments, which encompass the property of the rotation invariant. These properties were passed to a multi-layer fully-connected perceptron neural network with one hidden layer [53]. The weights of this network were trained using a back propagation algorithm, which is based on the generalized Delta rule [54].

For the ANN classifier, training set is based on the target library (Data Set A and B) in Appendix S1 and S2 respectively. For the Cross-plot technique, the same sets of pre-classified data are stored in its repository database. Experiments are conducted from a high-resolution video of the Dassault Rafale air target and the extracted images were received from this video (Data Set C). Here, *D_1_* is defined as the pre-classified data set for ANN and Cross-plot ATR, *D_2_* as the test data set, *P_TP_* as the percentage of objects correctly identified as positive targets (True-Positive), *P_FP_* as the percentage of objects incorrectly identified as positive targets (False-Positive), and *P_FN_* as the percentage of objects incorrectly identified as negative targets (False-Negative). The Precision is defined as the ratio of *P_TP_* to (*P_TP_* + *P_FP_*), and Recall is the ratio of *P_TP_* to (*P_TP_* + *P_FN_*). The Cross-plot has a higher precision and recall performance compared to the ANN method. This lends support to justification of the better performance by the Cross-plot pattern recognition technique.

It may be worthwhile noting that false-alarm and detection rate reduces as the number of nodes, *N* that is used in Cross-plot, or the number of hidden neurons, *n* in the hidden level of an ANN classifier increases. So to achieve a fair comparison of accuracy in pattern identification, suitable adjustments are made such that *N* = 8 and *n* = 5 such that the speed of processing is equal for both frameworks. The results based on their identification performance (Table 1) show that the ATR system based on Cross-plot technique has a lower false alarm rate and achieved a greater accuracy of detection as compared to the ANN based system.

**Table 1:** Classification performance of pattern recognition systems based on Cross-plot and artificial neural network.

Technique	*D_1_*	*D_2_*	*P_TP_* (%)	*P_FP_* (%)	*P_FN_* (%)	Precision	Recall
**Cross-Plot**	A	B	68.3	3.33	5.00	0.954	0.932
	A ∪ B	C	70.0	-	6.67	-	0.913
**ANN**	A	B	63.3	5.00	8.33	0.927	0.884
	A ∪ B	C	65.0	-	10.0	-	0.867

Pre-classified information is based on Data Set A (60 targets) for testing Data Set B (60 targets). Then, the combination of Data Set A and B (total 120 targets) is for testing Data Set C (60 positive targets) based on extraction of one target from every 5th frame of the video that consists of a total of 304 frames. Here, *D_1_* and *D_2_* are defined as the pre-classified and test data sets. The *P_TP_ P_FP_* and *P_FN_* are defined as the True-Positive, False-Positive, and False-Negative result classification. The Precision and Recall are equal to *P_TP_* over (*P_TP_* + *P_FP_*) and *P_TP_* over (*P_TP_* + *P_FN_*) respectively. Since Data Set C comprises of the same air target class and established as all positive target outputs, the False-Positive result classification is not applicable. Both Precision and Recall results are higher for the Cross-plot technique in comparison to its artificial neural network counterpart.

## Discussion

The proposed approach requires the generation of object signature from the data set and all the information about this item can be derived from the Cross-plot. The strength of this technique is the element of computational efficiency. The computation of the Cross-plot between the pattern and the node is of complexity *O*(*N + N*log*N*), where *N* is the number of data points. Because the comparison of the signature can be achieved without exhausting huge computational resources, this technique enables the possibility of the identification of targets in real time.

In shape retrieval systems, partial content information from a pattern is always extracted and condensed into a signature. The limitation of this approach becomes apparent when the structural details of the target are vague. This is because of the increase in perceptual similarity of the shapes pertaining to the binary target image as the air target is captured at greater distances. The loss of shape content information is determined by the resolution of the signature. The signature map is efficient in extracting the shape feature of binary image data with minimum computational and memory resources, and it is used as a thumbprint of the target in the database. Despite the many advantages, full information of the structural specifications of the target that is presented in its binary pattern cannot be contained. Nevertheless, storage space in the memory constrained database can be made available for more instances of training data to be added because of their low-memory signatures. The size of the signature is proportional to the duration for comparison, and can be made minimal at the expense of limited feature extraction and accuracy of identification. This signature can be scaled to an appropriate size depending on the memory space in the target reference database and an optimized accuracy of identification.

Comparison with pattern recognition systems based on different tools such as neural networks can be made. For the neural network-based recognition system [55], more neurons may be required to maintain the same accuracy of detection if the number of known targets with different shape contents increases. It is also difficult for the value of correlation to be indicated. Complexity of computation exceeds *O*(*N*), *N* is number of pixels that form the binary pattern. In addition, this model is not rotation, scaling, and translation invariant. When using the SCX (Solidity Eccentricity Extent) model, there is poor accuracy in identification of airplane silhouettes especially when there is region loss within patterns. Moreover, the poor accuracy in distinguishing airplanes that have close perceptual similarity makes the system inflexible. As there are no correlation metrics, the degree of matching is hard to indicate. The accuracy of identification cannot be varied with the speed of retrieval. The model is not noise, orientation, pixel-loss, and blurring invariant and patterns need to be preprocessed before classification.

For the shape context method, histogram of relative distances based on a log *r* versus *θ* grid serves a discriminative descriptor [56]. Analogously, consolidative count-of-points based on a Cartesian grid serves as the discriminative descriptor for the Cross-plot technique. Both approaches relies on global shape information into a local descriptor based on developing a set of vectors that express the configuration of the shape relative to a reference point. Therefore, the two methods work optimally only when comparing shapes derived from gray-scale images rather than from line representations. In addition, when implementing the shape context approach, reference points taken on a discontinuous contour of the shape as a result of region loss may not represent the true shape contour accurately. Sectional loss of shape parts violates the assumption that the sampled points are able to approximate the underlying continuous shape, and causes ambiguity in matching. Since the Cross-plot matching is independent of contour coordinates, an accurate discriminator can be achieved for shape objects that are subjected to distortion, noise, and region loss when using the same feature set. Therefore, the Cross-plot remains relatively robust for discontinuous shape recognition, whereas the shape context method will have failed for the above mentioned discrepancies.

The technique of using Cross-plots for pattern recognition surpasses many existing techniques due to its robust performance. The measurements of (i) area and perimeter, (ii) length of maximum dimension, (iii) moments relative to the centroid, (iv) number and area of holes, (v) area and dimensions of convex hull, (vi) number of sharp corners, (vii) number of intersections with a check circle, and (viii) angles between intersections, are some of the shape analysis techniques used in pattern recognition [9]. Most of these feature measurements will have failed for disconnected patterns such as that shown in Figure 7 (c) if the features extraction is not able to identify the overall shape characteristics despite such critical feature defects.

This paper presents the proof for using the Cross-plot as a potential tool for pattern recognition and motivates a number of open questions for further investigation. In particular, determination of the optimal resolution of the signature *vis-à-vis* the number of nodes and the resolution for each Cross-plot curvature that is sufficient to develop an accurate shape retrieval system running on low computational resources requires further investigations. The threshold of prediction failure based on incrementing the degree of pattern distortion for a specific configuration of signature is a challenge to the reliability of this proposed approach. Exploration of the effect of different datasets along with varying database sizes is beyond the scope of this proof-of-concept study, and is an interesting open question for future implementations. Other future works may include investigation of multiple distortion mechanisms such as a combination of shape transformation, noise and pixel loss on the accuracy of the target recognition system.

## Supporting Information

Appendix S1 **Data Set A – 6 sets of pattern variants with 10 stages of modification.**
(DOC)

Appendix S2 **Data Set B – 35 sample patterns from database of 60 targets.**
(DOC)

Appendix S3 **Pseudo-code for signature generation of a binary pattern.**
(DOC)
