# Supplementary material for: Automatic Target Recognition Based on Cross-Plot
Source: PLoS One. 2011 Sep 29;6(9):e25621. doi: 10.1371/journal.pone.0025621 (PMC3183066; doi:10.1371/journal.pone.0025621)
Supplement: Appendix S1 — Data Set A – 6 sets of pattern variants with 10 stages of modification. (DOC) [file pone.0025621.s001.doc]

**APPENDIX S1**

**Data Set A – 6 sets of pattern variants with 10 stages of modification**

| **Distortion** | **Pixel Loss** | **Scaling** | **Blurring** | **Rotation** | **Orientation** |
| --- | --- | --- | --- | --- | --- |
| 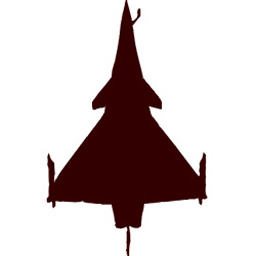 | 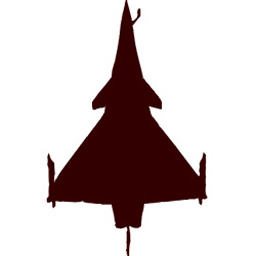 | 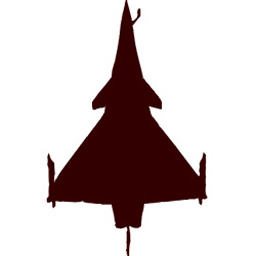 | 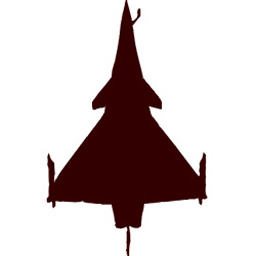 | 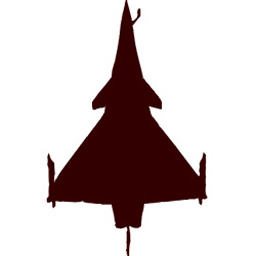 | 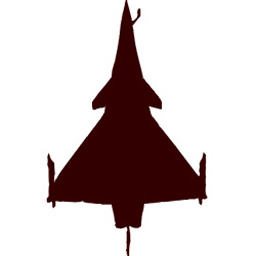 |
| 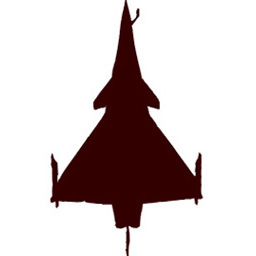 | 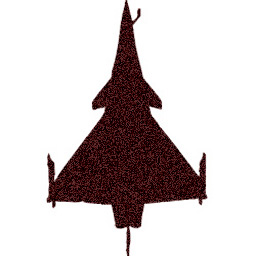 | 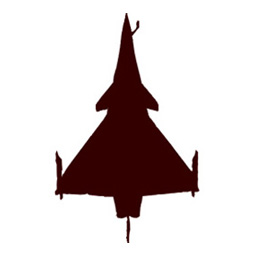 | 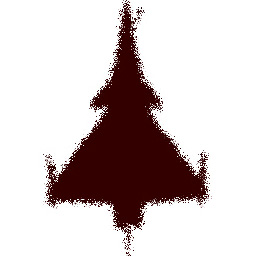 | 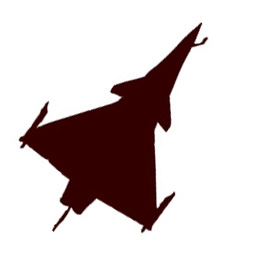 | 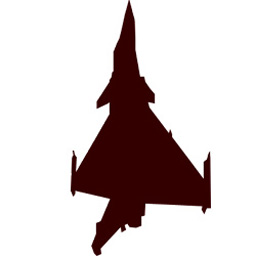 |
| 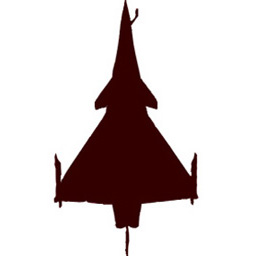 | 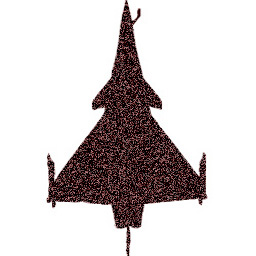 | 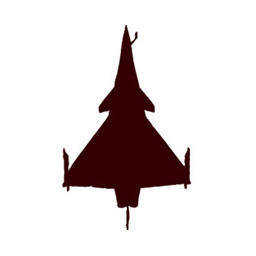 | 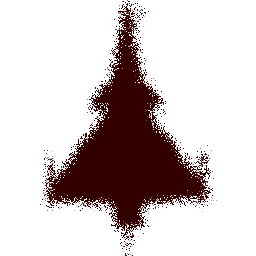 | 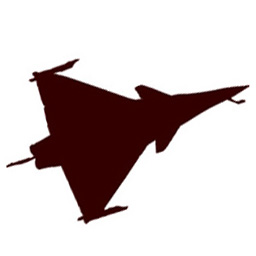 | 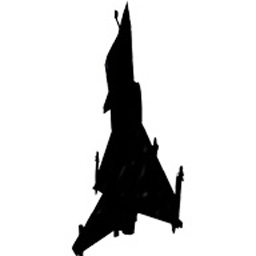 |
| 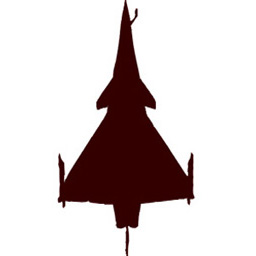 | 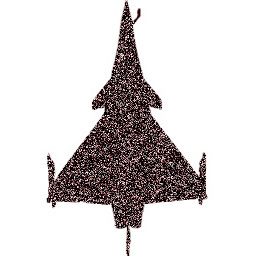 | 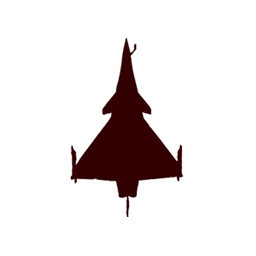 | 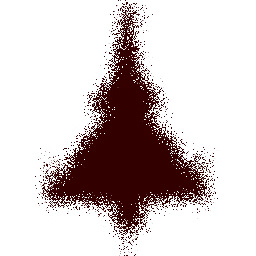 | 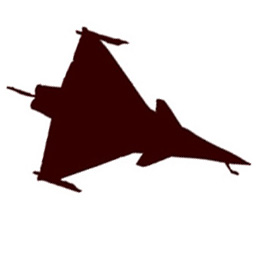 | 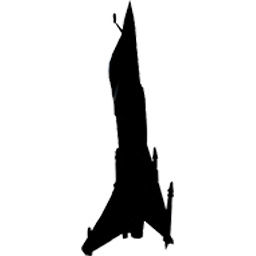 |
| 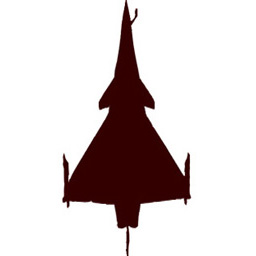 | 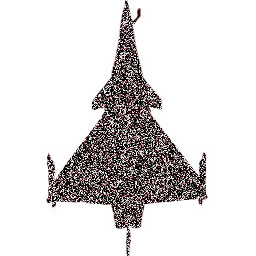 | 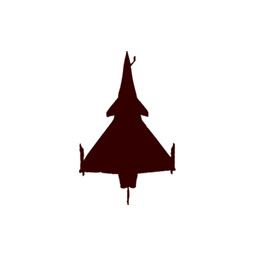 | 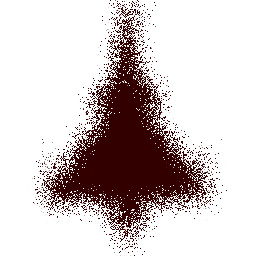 | 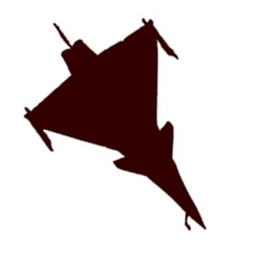 | 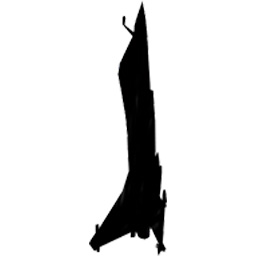 |
| 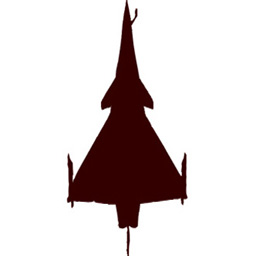 | 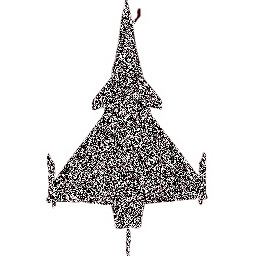 | 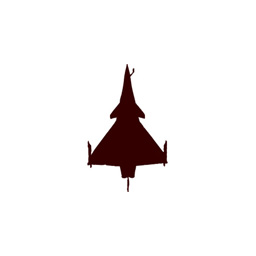 | 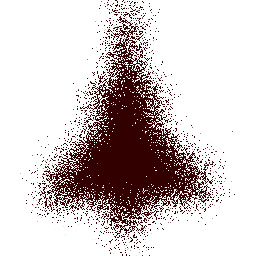 | 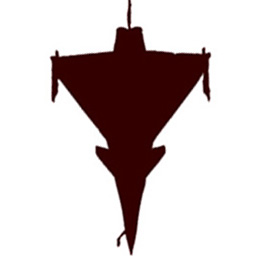 | 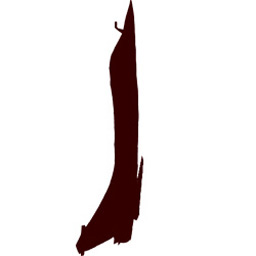 |
| 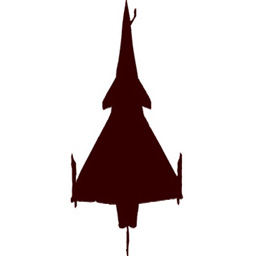 | 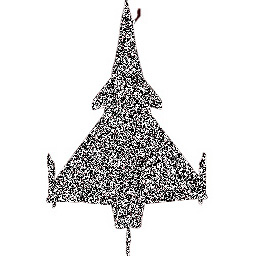 | 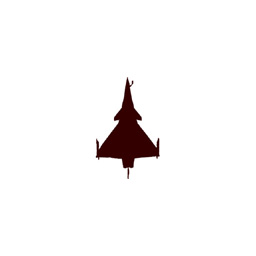 | 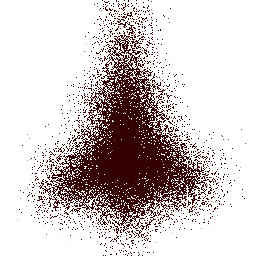 | 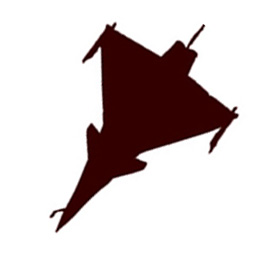 | 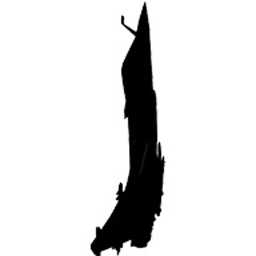 |
| 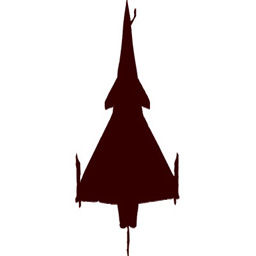 | 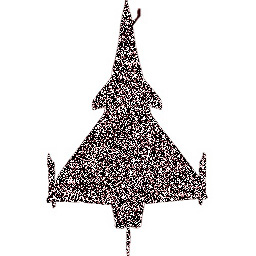 | 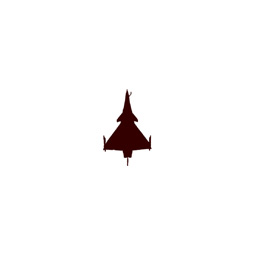 | 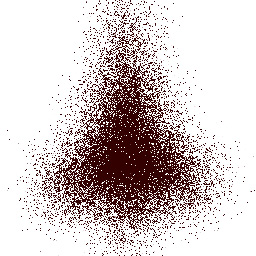 | 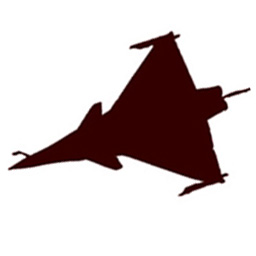 | 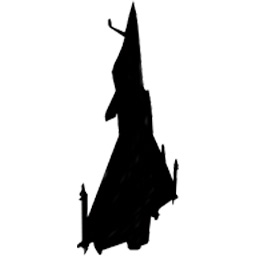 |
| 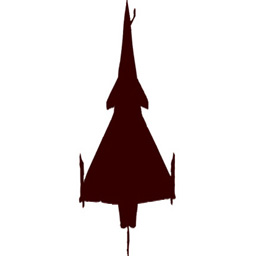 | 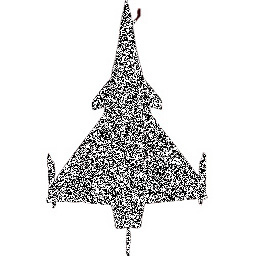 | 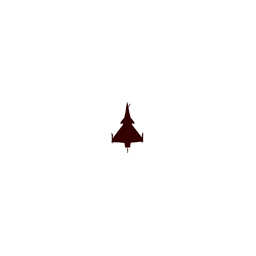 | 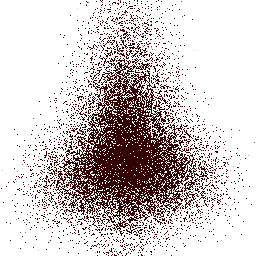 | 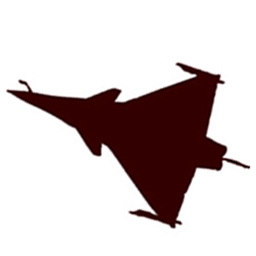 | 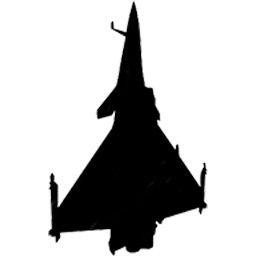 |
| 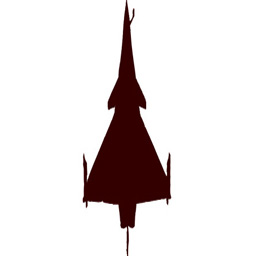 | 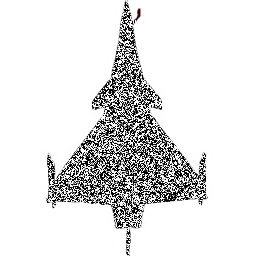 | 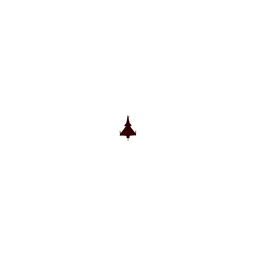 | 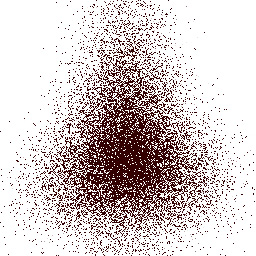 | 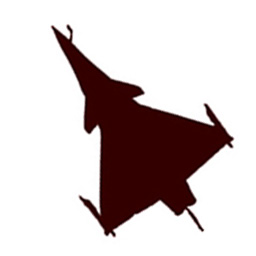 | 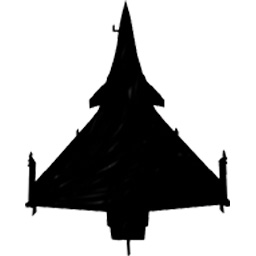 |
