# Supplementary figures and images for: Automatic Target Recognition Based on Cross-Plot
Source: PLoS One. 2011 Sep 29;6(9):e25621. doi: 10.1371/journal.pone.0025621 (PMC3183066; doi:10.1371/journal.pone.0025621)

**APPENDIX S2**

**Data Set B – 35 sample patterns from database of 60 targets**

| 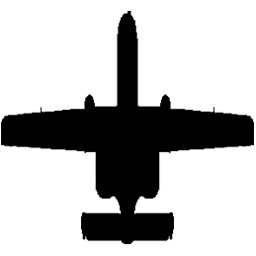 | 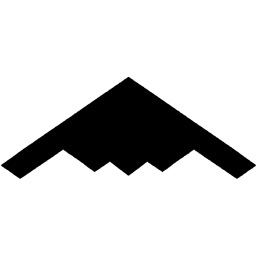 | 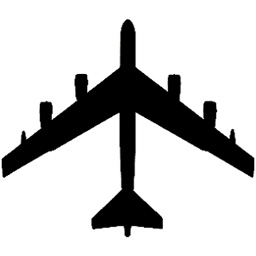 | 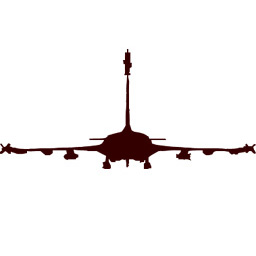 | 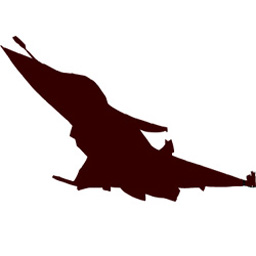 |
| --- | --- | --- | --- | --- |
| 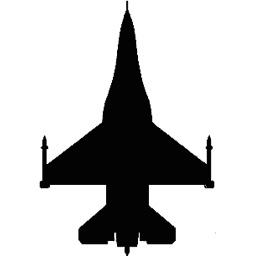 | 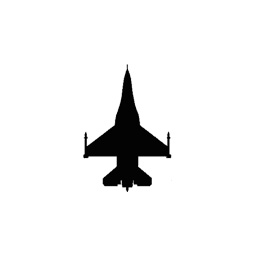 | 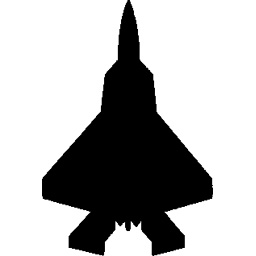 | 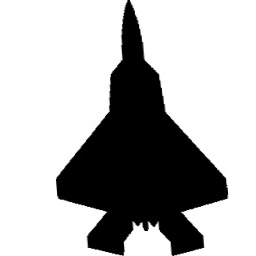 | 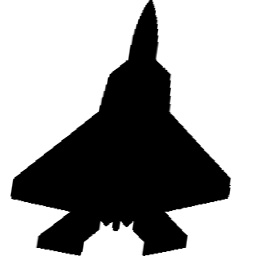 |
| 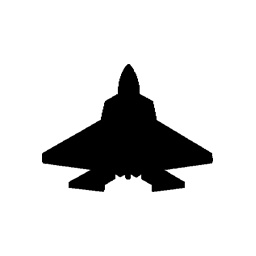 | 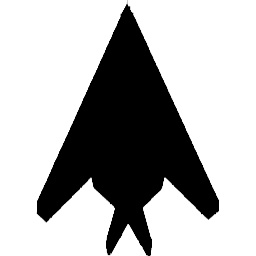 | 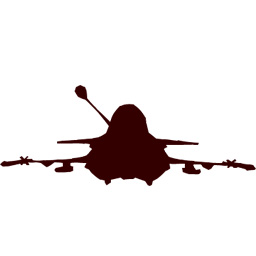 | 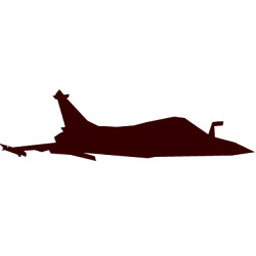 | 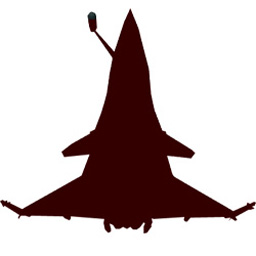 |
| 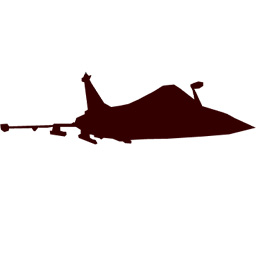 | 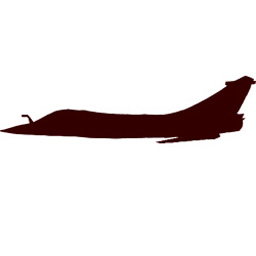 | 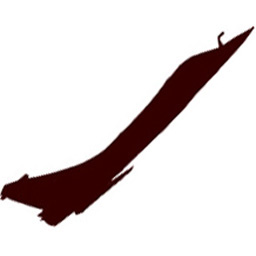 | 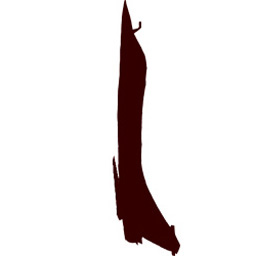 | 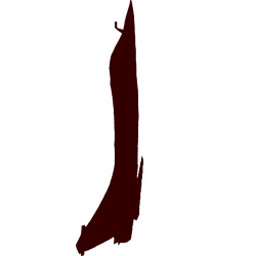 |
| 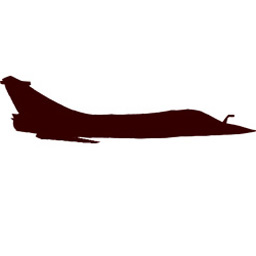 | 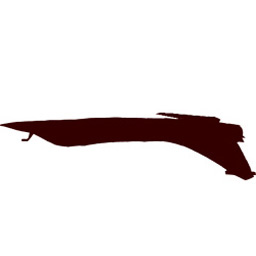 | 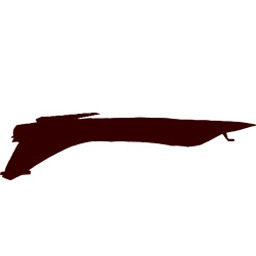 | 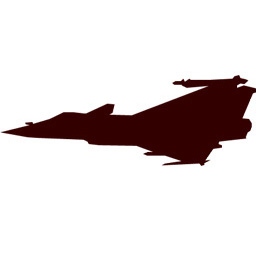 | 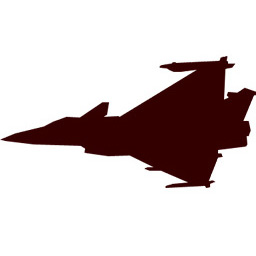 |
| 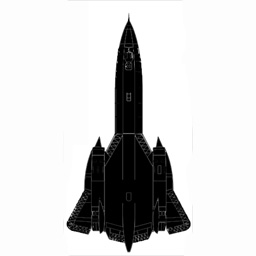 | 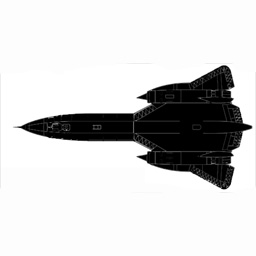 | 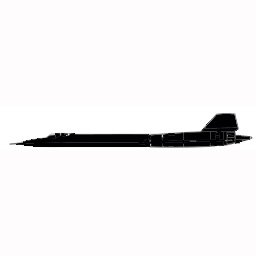 | 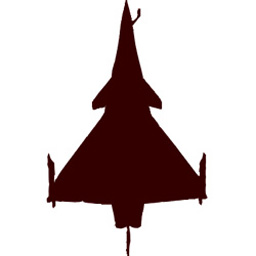 | 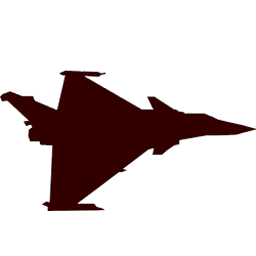 |
| 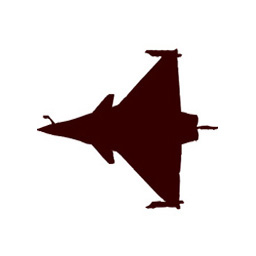 | 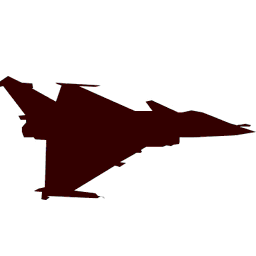 | 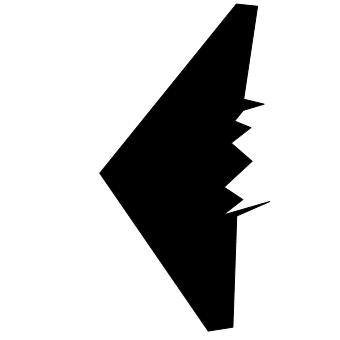 | 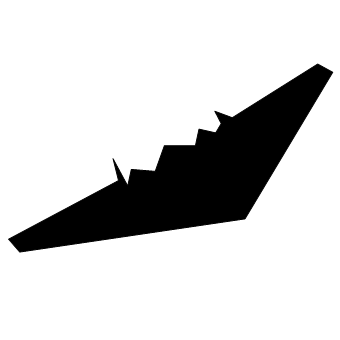 | 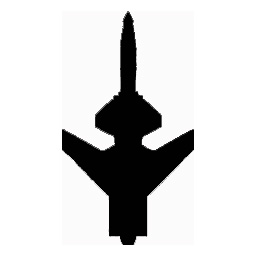 |

Supplement: Appendix S2 — Data Set B – 35 sample patterns from database of 60 targets. (DOC) [file pone.0025621.s002.doc]
