# Supplementary material for: Automatic Target Recognition Based on Cross-Plot
Source: PLoS One. 2011 Sep 29;6(9):e25621. doi: 10.1371/journal.pone.0025621 (PMC3183066; doi:10.1371/journal.pone.0025621)
Supplement: Appendix S3 — Pseudo-code for signature generation of a binary pattern. (DOC) [file pone.0025621.s003.doc]

**APPENDIX S3**

**Pseudo-code for signature generation of a binary pattern**

In this set of codes, the square brackets, [M] represents a matrix, M, and M(i) represents an array from row i of M. If [M] is an array, then M(i) is the (i)th entry of the array, M. [M, N] represents the column-wise concatenation of matrices M and N. Based on these definitions, the pseudo-code for signature generation of a binary pattern is given as follows:

1) Obtain spatial coordinates, Orig_DatSet of input target pattern.

2) Obtain the base pattern, New_DatSet from Orig_DatSet by performing SVD.

3) Determine the dimensional characteristics of the pattern such as its range of x-coordinates and y-coordinates, width and height, and the centre of area. These parameters are represented by minX, maxX, minY, maxY, xDist, yDist, centerX, centerY respectively.

4) Determine characteristics of reference nodes alignment by calculating the minimum circle boundary such that the diameter of the circle is the larger value of xDist and yDist under comparison. The following properties are calculated:

Radius = Diameter/2,

inc = Radius*sin(pi/4).

5) The positions of eight arbitrary reference nodes are calculated using the calculated properties. The coordinates of the nodes are represented in 8-by-1 arrays such that NodeX and NodeY consist of the X- and Y-coordinates of all the eight nodes:

NodeX = [(centerX-inc), (centerX), (centerX+inc), (centerX+Radius), (centerX+inc), (centerX), (centerX-inc), (centerX-Radius)],

NodeY = [(centerY-inc), (centerY-Radius), (centerY-inc), (centerY), (centerY+inc),

(centerY+Radius), (centerY+inc), (centerY)].

The two arrays are concatenated column-wise to form an 8-by-2 matrix representing the coordinates of the nodes:

NodeXY = [NodeX, NodeY].

1. Here, the function for calculating the Cross-plot of a node, P and data set, D is given by ‘Cross_plot(P, D)’ which returns an array, C of coordinates pertaining to the Cross-plot curvature. The number of radial increments is defined by a constant, NDiv. Sub-functions are defined to facilitate the Cross-plot generation. ‘getN’ gives the total number of pixel points in D. ‘getMin’ and ‘getMax’ give the minimum and maximum values of their input arrays respectively. ‘getR’ gives the array of distances between P and every point from D. ‘findArray‘ extracts and outputs an array of items from R that satisfy an equality or inequality condition. ‘getLength’ computes the number of values in an input array. ‘log’ gives the logarithm of its input. The pseudo-code of the function is as follows:

Function C = Cross_plot(P, D)

R = getR(P, D)

min_R = getMin(R)

max_R = getMax (R)

interval_R = (max_R - min_R)/ NDiv

N = getN(D)

C = [ ]

i = 1

For curr_R = min_R To max_R At increments of interval_R Do

C(i) = [log(curr_R/max_R), log(getLength(findArray(R <= curr_R))/N)]

i = i + 1

End For

Return C

End Function

1. The thumbprint representation or signature of the pattern set, New_DatSet is a matrix comprising of 8 columns and with rows consisting of num_divisions number of entries. It is denoted by S and computed as follows:

S = [ ]

For curr_Node = 1 To 8 Do

C = Cross_plot([NodeX(curr_Node), NodeY(curr_Node)], New_DatSet)

S = [S, C]

End For

Output S
